# Aging and Circadian Disruption: Causes and Effects

**DOI:** 10.18632/aging.100366

**Published:** 2011-08-22

**Authors:** Steven A. Brown, Karen Schmitt, Anne Eckert

**Affiliations:** ^1^ Department of Pharmacology and Toxicology, University of Zurich, Zurich, Switzerland; ^2^ Neurobiology Laboratory for Brain Aging and Mental Health, Psychiatric University Clinics Basel, Basel, Switzerland

**Keywords:** circadian rhythms, aging, fibroblasts, peripheral cells, sleep

## Abstract

The relationship between aging and daily “circadian” behavior in humans is bidirectional: on the one hand, dysfunction of circadian clocks promotes age-related maladies; on the other, aging *per se* leads to changes and disruption in circadian behavior and physiology. For the latter case, recent research suggests that changes to both homeostatic and circadian sleep regulatory mechanisms may play a role. Could hormonal changes be in part responsible?

## INTRODUCTION

Increasing evidence suggests that disruption of circadian clock function - either genetically or environmentally - can exacerbate a wide range of age-related pathologies, ranging from cataracts to cancer. An excellent review on this subject was published recently in this journal [1]. Equally relevant, however, and even more painfully obvious, is the impairment of circadian function that occurs as a natural process of aging. The German language has invented the term “*senile Bettflucht*” (literally, senile bed evacuation) to describe the difficulty that elderly individuals have in sleeping at night, and the early hour at which they rise. Indeed, one in four aged persons reports regular use of a prescribed sleep medication [2]. Since such medications treat only the symptoms and are also potentially addictive, the origins of this sleep disturbance are an important public health question.

Age-related sleeping difficulties are actually twofold. On the one hand, elderly individuals will rise and also go to bed an average of two hours earlier than young adults [3]. Secondly, their nighttime sleep is considerably more fragmented, and contains a much lower proportion of “deep” or slow-wave sleep (SWS) [4]. Whether these two phenomena are linked or independent remains a subject of debate. Underlying causes are a matter of speculation.

Recently, by using primary human fibroblast cells as a model system, our laboratories reported that serum-borne factors (i.e. hormones) could play a role in age-related circadian disturbances [5]. Rather than being a comprehensive review, this Perspective is an attempt to set our findings more explicitly within the context of other data in the field than was possible in the context of the original research communication.

## THE EXPERIMENT UNDER DISCUSSION

**Figure 1:**
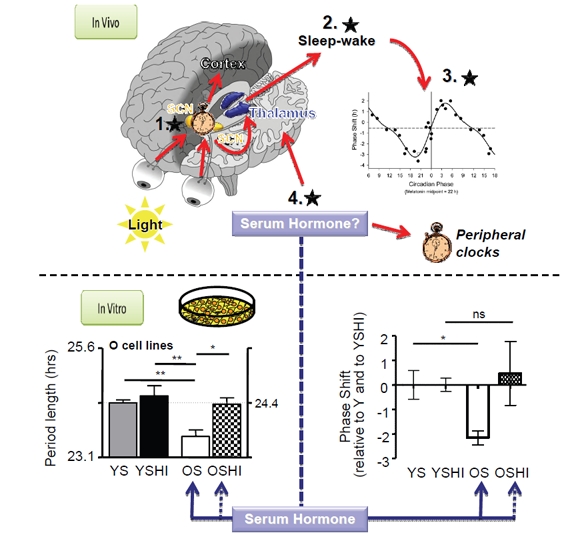
Top panel *In vivo*, a central clock in the suprachiasmatic nucleus (SCN) determines the timing of daily behavior, and communicates this timing to peripheral clocks in other tissues and brain loci that control sleep. The timing of sleep influences clock phase by controlling when the eyes receive environmental light. *Inset graph*, This light phase-shifts the clock differently at different times of day (Data reproduced from [11]). *Black stars*, Feedback loops affecting sleep in the elderly. 1. A shorter period in the SCN would shift sleep earlier, but this has not been observed experimentally in humans. 2. Changes to sleep-wake structure, either by affecting homeostatic sleep or by affecting the circadian drive to sleep at night, 3. could feed back to affect light availability and therefore clock phase because of natural time-dependent differences in phase shifting. 4. Hormones could directly affect peripheral clocks at sleep-wake centers to affect sleep timing without altering the central clock in the SCN. **Bottom panel**.*In vitro*, treatment of primary human fibroblasts with serum from older subjects (OS) results in a shorter period and an earlier phase of cellular circadian rhythms than that obtained with serum from younger subjects (YS). Heat treatment (OSHI) abolished this effect. (Data reproduced from [5].)

Exploiting the fact that human circadian clocks are conserved in most cell types, Pagani et al. examined the circadian properties of primary fibroblasts from older and younger individuals. Although the cells from both groups showed identical circadian properties (period, amplitude, entrained phase) when cultured identically, inclusion of serum from older individuals resulted in a shortening of circadian period and an earlier entrained phase in either cell type. This change was likely due to a substance in the serum of older individuals, because heat treatment gave older persons' sera the circadian properties of younger persons' sera, but did not change the properties of younger persons' sera (Figure 1, bottom) [5].

## BACKGROUND

In principle, sleep is regulated by two separable processes: a circadian one, which pushes diurnal species such as humans to sleep preferentially at night; and a homeostatic one, by which sleep drive increases with increasing time spent awake [6].

The circadian process is driven by a biological “circadian” clock. In mammals, the central clock controlling diurnal behavior is located in the suprachiasmatic nucleus of the brain hypothalamus (the SCN). Its mechanism is cell-autonomous, and is duplicated in “slave” clocks in nearly all the cells of the body. The molecular mechanism of this clock has been reviewed previously, including in this journal [1]. Numerous studies have demonstrated that its genetically encoded period length (the time taken for one complete cycle under constant conditions) directly affects the phase of human behavior and gene expression: individuals with longer periods have a later phase than those with shorter periods, looking either at human behavior or at gene expression [7-9].

In mammals, entrainment of the circadian clock to its environment is driven predominantly at the ocular level by environmental light. Hence, blind individuals with an endogenous clock period significantly different from 24 hours are unable to synchronize to the solar day [10]. The response of the clock to light is asymmetric, and light at different times of the day or night will shift the clock in different directions and by different amounts (Figure 1, bottom inset). As one might predict, evening light delays the clock, and morning light advances it [11].

The homeostatic process is much less well understood at the molecular level, but may be a fundamental property of neural assemblies [12]. It involves global synchronization of rhythmic thalamocortical firing patterns whose hallmark is a predominance of particular frequencies measured by EEG. Sleep is divided into different “stages” characterized by different frequency bands, and an individual will typically alternate among these stages in a defined pattern for several episodes during the night. The “intensity” of sleep is determined by time spent awake, by genetic factors, and by environmental disturbance, with more profound sleep characterized by a greater intensity of these EEG frequencies, indicative of more pervasive neuronal synchrony [13].

## THEORY

Purely theoretically, based upon the mechanisms outlined above, numerous hypotheses can be advanced to explain the disruption of sleep in the elderly. Let us consider the two features of this disruption separately. The earlier bedtime and waking time of elderly individuals could be a result of a shortening of endogenous circadian period. It could also arise from a change in the way the clock changes phase in response to light: anything that resulted in a net gain of morning light or loss of evening light would result in an earlier phase. Finally, the early phase of elderly individuals could arise from homeostatic effects: an increased sleep need would advance bedtime or delay wake time, and a decreased sleep need would advance wake time or delay bedtime.

The second property of sleep in the elderly is its fragmentation. Lower circadian amplitude would result in greater difficulty in sleeping at night, and greater ease of napping. Alternatively, homeostatic processes could play a role: lower homeostatic sleep drive would also result in sleep fragmentation. Greater susceptibility to environmental disturbance would have the same effect.

By imagining age-related changes to both homeostatic and circadian mechanisms, it is possible to rationalize separately the earlier phase and the increased fragmentation of sleep that occur in elderly individuals [14]. However, explaining both effects with the same hypothesis is not simple. One idea is that dampened circadian amplitude results in sleep fragmentation at night and daytime napping [15]. These changed sleep patterns would be reinforced by changes in the timing of light, which shifts phase earlier [16]. A second hypothesis suggests that reduction in homeostatic sleep drive could accomplish the same effect, fragmenting sleep directly and shifting phase via altered light choice [17]. These models are shown schematically in Figure 1.

## OBSERVATIONS

Although numerous behavioral studies have been conducted over the past decade to address these hypotheses, no clear picture has emerged. Evidence to support and contradict each of them exists: Circadian period length: Although a shortened behavioral period length as a consequence of age has been observed in some animals [18], careful studies of older humans under conditions of “forced desynchrony” show no hint of such changes. In these experiments, subjects were kept under photoperiods so long (28h) that their endogenous circadian clocks could not adjust. Circadian period was determined under these “free-running” conditions by measuring rhythmic expression of the hormone melatonin, or diurnal variation in body temperature [14]. Pagani et al. showed shortening of period in human fibroblasts, but only in the presence of blood serum from older individuals [5].

Phase shifting: In humans, phase shifts in response to very bright light do not differ significantly between older and younger subjects, at least for phase delays [19, 20]. Phase advances were attenuated in some studies [19, 21], but this data would not explain *earlier* phases in older individuals. Moreover, these studies used very bright light to obtain maximum phase-shifting. Whether these results can be generalized to dimmer light remains an open question, because considerable reduction in lens transmission occurs with age [22], and reduced phase delays in response to moderate light have been reported [23].

Circadian Amplitude: Changes in circadian amplitude are more difficult to measure. Certainly, melatonin production has been shown in multiple studies to diminish with age [24], but it is not clear that this reflects a change in circadian amplitude *per se*: size and calcification of the pineal gland that produces melatonin also diminish with age [25, 26]. Circadian rhythms of body temperature also decline with aging, but these are in part activity-determined [27].

Sleep fragmentation: Changes in sleep patterns in the elderly have been well-documented, but ascribing them specifically to circadian or homeostatic changes are more difficult. For example, a tendency toward shallow, fragmented sleep could be explained by a weakened circadian arousal signal at that time [15]. Surprisingly, recent studies suggest that older adults have *less* daytime sleep propensity than younger ones [4]. At the same time, total sleep duration is reduced, *and* sleep fragmentation increases. These results imply effects upon homeostatic control -- specifically, a reduction in sleep need has been documented in elderly individuals [28], accompanied by a reduction in sleep efficiency. Partly contradicting this, the response to low sleep pressure in laboratory conditions is similar in younger and older individuals, suggesting an interplay between circadian and homeostatic effects [29].

## UNCERTAIN CONCLUSIONS

So far, little evidence exists to suggest that the period length of circadian behavior is changed in elderly individuals. Moreover, although studies suggest that homeostatic sleep is affected in fundamental ways in older individuals, these observations are likely insufficient to explain the marked circadian changes observed. In forced desynchrony studies that showed increased sleep fragmentation, investigators also observed an earlier sleep onset relative to the phase of the hormone melatonin [14]. Similarly, under constant routine studies under constant dim light, the phase of gene expression in blood cells is still advanced [30]. Since the light cycle in these studies was *not* systematically affecting the circadian clock, these results imply that changes in circadian phase are unlikely to be explained via strictly homeostatic mechanisms affecting light choice. Of course, homeostatic sleep mechanisms might also have more direct effects upon the circadian oscillator [31, 32], but these mechanisms remain to be explored.

Against this context, Pagani *et al.* postulated that hormonal changes in elderly individuals could alter circadian period in peripheral cells. In an environment entrained by the solar day, such changes would easily translate into changes in phase. These observations do not, however, explain age-related changes in circadian amplitude or in homeostatic sleep. Moreover, such an explanation presumes that nuclei in the brain directing sleep timing are affected by this hormone, but that the master clock in the suprachiasmatic nuclei is not (since no corresponding changes in human behavioral period have been documented). Thus, it is at best a partial explanation. What makes it attractive is that hormonal changes offer the likely possibility of pharmacological remedy.

## FUTURE DIRECTIONS

This Perspective has confined itself (mostly) to discussion of specific theories about the interplay among aging, sleep, and the circadian clock. Already, mutation and gene profiling studies have implicated specific clock genes in the ageing process [1, 33, 34]. In fact, however, the best evidence for any model comes in the form of detailed mechanisms, and here is undoubtedly where future research will be directed. For example, in rodent models, aging is correlated with losses of specific classes of neurons (orexin and CRH) that could affect sleep architecture [35]. Experiments to address whether these changes are necessary and sufficient to explain fragmented sleep -- and whether similar changes are observed in the aged human brain that correlate with sleep disturbance - will reinforce homeostatic models. Similarly, it is well-known that human aging is accompanied by large alterations in hormone balance, both in the hypothalamic-pituitary-adrenal axis and elsewhere [36]. If Pagani *et al.* wish to suggest that a hormone is in part responsible for age-related circadian dysfunction, then the best evidence in their favor would be identification of the suspected factor and characterization of its effects.
